# The emerging role of skeletal muscle as a modulator of lipid profile the role of exercise and nutrition

**DOI:** 10.1186/s12944-022-01692-0

**Published:** 2022-08-30

**Authors:** Tzortzis Nomikos, Spyridon Methenitis, Demosthenes B Panagiotakos

**Affiliations:** 1 Department of Nutrition and Dietetics, School of Health Sciences & Education, Harokopio University, Athens, Greece; 2 Sports Performance Laboratory, School of Physical Education and Sports. Science, National and Kapodistrian University of Athens, Athens, Greece; 3 Theseus, Physical Medicine and Rehabilitation Center, Athens, Greece

**Keywords:** Skeletal muscle, Lipid profile, Exercise protocols, Muscle fibers, Intramuscular triglycerides, Dyslipidemia

## Abstract

The present article aims to discuss the hypothesis that skeletal muscle per se but mostly its muscle fiber composition could be significant determinants of lipid metabolism and that certain exercise modalities may improve metabolic dyslipidemia by favorably affecting skeletal muscle mass, fiber composition and functionality. It discusses the mediating role of nutrition, highlights the lack of knowledge on mechanistic aspects of this relationship and proposes possible experimental directions in this field.

## Introduction

To date, numerous studies have proven the deleterious impact of poor lifestyle choices and unhealthy body composition on cardiometabolic risk factors, including dyslipidemia [1, 2]. Lately, the metabolic properties of skeletal muscle and their association with its morphology and functionality have attracted the interest of many researchers and health professionals for their role in obesity, insulin resistance, metabolic syndrome and diabetes. For example, it has been reported that the metabolic expenditure of lean body mass (LBM)—and not nutrition itself—is adolescents' primary determinant of body composition, as both normal weight and obese adolescents tend to receive increased total energy intake while at the same time exhibiting poor nutritional habits [3]. Muscle mass, the composition of muscle fibers and their functionality are now identified as crucial determinants for the development of metabolic pathologies and as potential therapeutic targets against them [4–9]. However, although skeletal muscle is the largest tissue of the human body (~ 40% of body weight) and the main organ of fatty acid oxidation, either during rest but mostly during exercise, its role in lipid metabolism is underappreciated [10]. Therefore, the present article aims to discuss the hypothesis that skeletal muscle per se but mostly its muscle fiber composition could be significant determinants of circulating lipids, lipoproteins and lipid metabolism and that certain exercise modalities may improve metabolic dyslipidemia by favorably affecting skeletal muscle mass, fiber composition and functionality.

### The unappreciated role of skeletal muscle and muscle fiber composition

The skeletal muscle is composed of three types of muscle fibers, namely, muscle fibers Type I (slow contracting, low force-power production, highly fatigue-resistant fibers, large oxidative metabolism), Type IIa (fast contracting, moderate force-power production, moderate fatigue-resistant fibers, large oxidative and glycolytic metabolism) and Type IIx (very fast contracting, highest force-power production, fatigue-prone fibers, only glycolytic metabolism). The three types of muscle fibers have distinct contraction velocities and metabolic potential due to different contractile protein isoforms and different oxidative/glycolytic enzyme profiles [8, 11]. The specific metabolic/mechanical phenotype of each muscle fiber type in combination with its size and its relative percentage in the skeletal muscle tissues determines the size, metabolic and mechanical abilities of skeletal muscles [11–15]. In the major human locomotor muscles, such as the gastrocnemius and the quadriceps, approximately half of the muscle fibers are type I, although this percentage may vary between 80 and 20% [16]. However, abnormal muscle fiber composition, with extremely low proportions of Types I and IIa in favor of Type IIx muscle fibers, accompanied by low lean body mass (LBM) and high fat-to-lean mass ratios, are commonly observed in middle-aged/elderly individuals with obesity, diabetes, metabolic dysfunctions and unhealthy glycemic-lipidemic blood profiles [13, 17–22]. This skeletal muscle phenotype has been linked with poorer body composition, dyslipidemia and increased risk for cardiovascular diseases [6, 17, 18, 23, 24]. Until recently, it was a common belief that the observed alterations in skeletal muscle mass and fiber type composition were the result of low-grade chronic inflammation and oxidative stress, as well as of the metabolic dysfunctionalities induced by cardiometabolic diseases, which negatively affected the oxidative capacity of muscle fibers, especially Types I and IIa, and thus led to their reductions, while Type IIx muscle fibers remained unaffected by these issues [25].

However, it has been recently proposed that fiber type composition per se may also have a significant contribution to the development of cardiometabolic diseases [9], but until now, only indications exist to support this suggestion. For example, endurance athletes are characterized by a high % of cross sectional area (%CSA) occupied by Type I and IIa fibers and a low %CSA occupied by Type IIx fibers [15, 26], while they consume significantly higher amounts of fatty acids than the recommended [27–31] and have increased or at least the same intramuscular lipid concentrations compared to nonathletic, obese and diabetic populations [32]; however, they have better insulin sensitivity [33], lower Total Cholesterol (Chol), Triglycerides (Trig), Low-Density Lipoprotein blood (LDL) concentrations and greater High-Density Lipoprotein (HDL) concentrations [28, 29, 31] compared to sedentary, obese and insulin resistance patients [34]. This “athletes’ paradox” phenomenon has been attributed to the greater metabolic stress during their regular training but mostly to the increased oxidative capacity of athletes’ skeletal muscles partly due to the increased proportion and enhanced metabolic properties of their Type I fibers, properties that are absent in nonathletic, obese and diabetic populations [33]. In support, it has also been suggested that the increased accumulation of intramuscular lipids in obese individuals derives basically from metabolic inflexibility, especially of Type I, IIa fibers, for fat oxidation, compared to fat uptake per se [34, 35]. In contrast, it has been suggested that athletes’ oxidative muscle fibers, with their training-induced robust oxidative capacities, act as a “protective mechanism” against the lipotoxicity of intramuscular lipids and its resulting insulin resistance and dyslipidemia [19, 32, 33, 36]. Furthermore, the individuals' responses to nutrition seem to depend, among others, on the metabolic activity of muscle tissue and thus on muscle fiber type metabolic properties [37]. From a very limited number of interventional studies on animals [38–40] and humans [22, 41, 42], it seems that the initial increased size, proportion and metabolic properties of their oxidative muscle fibers were associated either with the lowest increases in body/fat mass and a lower risk for the development of dyslipidemia during long-term high caloric/high fat diets or with greater beneficial changes in their body composition and glycemic and lipidemic blood profiles after a period of hypocaloric diets. Again, these observations were attributed to the “protective mechanism” of oxidative muscle fibers [34, 41]. In simple words, according to the above reports, it seems that obesity-resistant animals and humans are characterized by increased proportions and metabolic functionality of Type I muscle fibers, while obesity-prone ones are characterized by increased proportions of type IIx/IIb muscle fibers, while the negative or positive effects of nutrition on their body composition and glycemic-lipidemic profiles seem to be controlled by their fiber type composition [22, 38–42]. These observations may also, at least partly, contribute to the interindividual differential responses to nutrition. According to the above reports, it seems that it is not the unhealthy nutrition per se that affects body composition and lipidemic profiles but how humans and animals metabolize their nutritional intake, with their muscle fiber composition to possess a pivotal role on this, but this must be verified by long-term randomized, controlled studies. Along with previous reports, it has been recently reported that at least very active, young healthy females whose skeletal muscles are characterized by a high %CSA of type I, IIa and a low Type IIx %CSAs are also characterized by lower Chol, Trig, LDL and greater HDL concentrations, in contrast to those with significantly increased Type IIx %CSA, even if no significant differences in their nutritional intake were found [12]. Furthermore, a 10-week resistance training protocol induced beneficial changes in the lipid profile, which were highly correlated with the changes in muscle fiber composition. Specifically, individuals experiencing the greatest reduction of their Type IIx fibers and the highest increments of their Type I, IIa %CSAs were those showing healthier changes in their lipidemic profiles [12]. Finally, individuals with a high LBM show a higher capacity to oxidize fatty acids, increased energy expenditure, greater ability to clear triglycerides from the circulation and healthier body composition than those with a lower LBM [3, 4, 12]. Considering all the above, it could be hypothesized that the qualitative characteristics of skeletal muscle, especially its muscle fiber composition and metabolic properties, may have a broader, more sophisticated role in lipid metabolism than that which has been given to them until now.

### Gaps in our knowledge – future directions

As presented in the above paragraphs, skeletal muscle composition and functionality seem to have a pivotal role in the lipidemic profiles. Unfortunately, the majority of these reports come from observational studies, mainly in middle-aged and older individuals with cardiometabolic risk factors, and only scarce data exist for other populations or from interventional (nutritional or training or combination of them) studies. Moreover, well-designed exercise interventions, able to modulate skeletal muscle composition and functionality, should be conducted where the lipid profile will be monitored with the measurement of classic and novel lipid parameters, including lipoprotein profile, Lp(a), cholesteryl ester transfer protein (CETP), lecithin:cholesterol acyltransferase and HDL functionality, among others. Interestingly, it is still unknown whether the genetically determined increased proportion and size of Type IIx fibers could contribute to a worse lipid profile [9]. The identification of the cause-result relationships between fiber type distribution, dyslipidemia and lipid disorders should provide a better understanding of the in-depth pathophysiology of lipid metabolism and may identify muscle fiber type distribution as another physiological parameter that could be used as a prognostic parameter and perhaps as a risk factor for the development of dyslipidemia in the future.

However, the lack of biochemical and physiological mechanisms underlying the observed relationships between skeletal muscle and lipid metabolism is the main gap in our understanding of these relationships. Until now, only suggestions have been made for the explanation of these relationships, which mainly focused on fiber type-dependent metabolic properties and intracellular signaling. Type I followed by Type IIa fibers are characterized by an increased capacity of oxidative metabolism, especially of fatty acids, mitochondrial metabolism, higher ratios of mitochondria and capillaries per fiber, increased diffusion rates, greater functionality of molecular pathways such as reactive oxygen species (ROS), AMP-activated protein kinase (AMPK) and peroxisome proliferator-activated receptor gamma coactivator 1 alpha (PGC-1a) cascades, which are involved in cellular energy homeostasis, mitochondrial function, transportation-phosphorylation-oxidation of fatty acids/lipids into muscle fibers and metabolic procedures that are limited or absent in Type IIx muscle fibers [4, 7, 8, 43–50]. Furthermore, it was proposed many years ago that oxidative muscle fibers contribute to the greater clearance of LDL and VLDL from the blood compared to glycolytic fibers, as the concentrations and activities of major enzymes linked with the clearance of triglycerides from the circulation and the translocation of fatty acids into skeletal muscle (lipoprotein lipase, LPL, fatty acids binding and transport proteins) are muscle fiber dependent (I > II) [51–53]. Whether the above-mentioned different functionalities of fiber type intracellular cascades could affect lipid metabolism and blood lipid concentrations and explain the observed relationships between muscle fiber composition and lipidemic profiles, as well as the relationships between their training-induced adaptations, is still unknown. Furthermore, it is not known whether each type of muscle fiber differs in the concentration and activity of other enzymes related to muscle lipid/FFA metabolism, such as phospholipases, cyclooxygenases and lipoxygenases. Finally, according to the above, it is still unclear which is the “protective mechanism” of oxidative muscle fibers, which many researchers have suggested [19, 32–34, 36, 41]

Skeletal muscle cross-talk with other metabolic organs (adipose tissue, liver) enhances the metabolic response of the human body to exercise and favoring beneficial metabolic adaptations through the secretion of myokines, myobolites, myomiRNAs, extracellular vesicles and exosomes [4, 5, 7, 54, 55]. However, it is still debatable whether this cross-talk is fiber-dependent and to what extent it may affect circulating lipid concentrations.

From a more clinical point of view, it is not known whether different exercise modalities, through their ability to change the composition and functionality of skeletal muscle, could differently affect lipid metabolism. Our group has recently shown that individuals experiencing the highest reductions in Type IIx %CSA and concomitant increases in Type IIa %CSAs are those with the greatest improvements in their lipid profile after 10 weeks of resistance training [12]. Indeed, as muscle is a highly plastic tissue [45, 56–60], its properties can be changed according to nutrition but mostly to training-induced metabolic, physiological and mechanical stress. Increased training volumes upregulate molecular pathways controlling the size, type and metabolic procedures of muscle fibers, which ultimately results in the transmission of Type IIx to the more metabolically efficient Type IIa muscle fibers [4, 5, 7, 12, 45, 56], changes that are highly related to those of lipidemic profiles [12]. However, to date, we do not know the exact physiological-biochemical background of these observations. We also do not know whether there is a cause-effect relationship or whether they are just two concomitant, independent adaptations to training-induced stimuli. Furthermore, we do not know if different exercise modalities, through their distinct ability to affect muscle morphology, could differentially affect the general human body’s lipoprotein metabolism, including hepatic biogenesis of HDL particles [52]. Furthermore, as the majority of patients with dyslipidemia receive standard hypolipidemic drug schemes, it is unknown whether the combination with exercise training may enhance or limit their independent or combined effects on lipids metabolism and on training induced changes in muscle morphology, fiber type composition and metabolism. and why. Finally, it is not known whether exercise training can be personalized according to the type of dyslipidemia since different metabolic disturbances characterize each of these types.

Finally, the role of nutrition and diet on the interplay between skeletal muscle and lipid metabolism is very important but still ill-defined. A significant question that arises is whether long-term high caloric/high fat diets may alter muscle fiber composition and metabolic properties even in obesity-resistant individuals (which are characterized by increased proportions and metabolic functionality of type I muscle fibers). For example, the observed abnormal low oxidative, high glycolytic muscle fiber proportions and the low metabolic functionality of muscle fibers in obese individuals is commonly attributed to long-term poor, high caloric but mostly high fat (not ketogenic) nutrition [9, 25]. However, this has never been verified by good quality randomized, long-term intervention studies, especially in humans. The scarce data that exist until now basically come from animal studies. It has been reported that a long-term (12 weeks) but not short-term (4 weeks) high-fat diet induces significant increases in glycolytic muscle fibers in mice [61], while long-term high-fat diets induce significant negative changes in muscle fiber composition (decreases in oxidative and increases in glycolytic fibers) and metabolic properties mainly in male mice [62]. However, until now, at least to our knowledge, there has been a strong lack of data in humans. In addition, it is not known whether diets and/or supplements, which are commonly suggested to patients with hyperlipidemia, enhance or compromise the training-induced adaptations that are needed for the improvement of the lipid profile of these patients [63].

## Conclusions

The current article highlights the hypothesis that skeletal muscle, but mostly the distribution and the metabolic properties of muscle fibers, may play a crucial role in the regulation of lipid metabolism and lipidemic blood profiles. We also highlight the notion that exercise-induced changes in muscle fiber composition and metabolic capacities may be a promising therapeutic target against dyslipidemia, as has been recently suggested for Type 2 diabetic patients [7]. It also emphasizes the lack of knowledge on this issue, especially on the molecular mechanisms that may underlie these observations. Therefore, we strongly believe that the study of the regulatory role of skeletal muscle mass, morphology and functionality on lipid metabolism is a promising research field, and novel therapeutic targets and lifestyle intervention for dyslipidemia may arise.

## Data Availability

Not applicable.

## Abbreviations

%CSA: Percentage of the cross-sectional area of muscle occupied by each muscle fiber type
CSA: Cross-sectional area
HDL: High Density Lipoprotein
HFD: High-fat diets
LBM: Lean body mass
LCAT: Lecithin-cholesterol acyltransferase
LDL: Low density lipoprotein
TAG: Triglycerides
TC: Total cholesterol
VLDL: Very low density lipoprotein
